# Supplementary material for: Flexible metal-organic framework films for reversible low-pressure carbon capture and release
Source: Nat Commun. 2025 Aug 4;16:7135. doi: 10.1038/s41467-025-60027-6 (PMC12321995; doi:10.1038/s41467-025-60027-6)
Supplement: Supplementary file 1 — Supplementary Information [file 41467_2025_60027_MOESM1_ESM.pdf]

Supplementary Information

Flexible metal-organic framework films for reversible low-pressure carbon capture and release

Sumea Klokic,[1*] Benedetta Marmiroli,[2] Giovanni Birarda,[3] Florian Lackner,[4] Paul Holzer,[2] Barbara Sartori,[2] Behnaz Abbasgholi-NA,[5] Simone Dal Zilio,[5] Rupert Kargl,[4] Karin Stana Kleinschek,[4] Chiaramaria Stani,[1] Lisa Vaccari,[3] Heinz Amenitsch[2*]

[1] CERIC-ERIC, S.S. 14, 163.5 km in AREA Science Park 34149 Basovizza Trieste, Italy.

[2] Institute of Inorganic Chemistry, Graz University of Technology, 8010 Graz, Austria.

[3] Elettra Sincrotrone Trieste, S.S. 14, 163.5 km in AREA Science Park 34149 Basovizza Trieste, Italy.

[4] Institute of Chemistry and Technology of Bio-Based Systems, Graz University of Technology, 8010 Graz, Austria.

[5] IOM-CNR, Laboratorio TASC, S.S. 14, 163.5 km, Basovizza, Trieste 34149, Italy.

*** Correspondence** to sumea.klokic@elettra.eu and heinz.amenitsch@tugraz.at

Contents

1. Linker solutions pH determination 7

2. Integration of GIWAXS pattern 7

3. Photo-illumination protocol for monitoring the CO_2 uptake/release with QCM-D 8

4. SEM micrographs for Zn_2L_2DABCO film structures 9

5. IR and GIWAXS characterization of the $Zn_2(NH_2)_2$-BDC_2DABCO system 11

6. Isostructural growth of heteroepitaxial Zn_2L_2DABCO films (L = BDC, Me-BDC, MeO-BDC) 13

7. Orientation analysis of heteroepitaxial Zn-MOF films 17

8. CO_2 uptake by Zn_2L_2DABCO films (L = BDC, Me-BDC, MeO-BDC) 18

9. CO_2 uptake by Zn-MOF films (T = 295 K) 24

10. Azobenzene (AB) infiltration in Zn_2BDC_2DABCO, Zn_2MeO-BDC_2DABCO and Zn_2Me-BDC_2DABCO films 26

11. Calculation of AB loading level in Zn_2MeO-BDC_2DABCO and Zn_2OMe-BDC_2DABCO film structures 30

References 31

Figure S1 Sketch of GIWAXS data interpretation. Integration of 2D detector pattern was performed in the out-of-plane and the in-plane direction as denoted by the red dotted line, which corresponds to a cut margin of 20 pixels. 7

Figure S2 SEM images of the Zn_2L_2DABCO film structures and sketch of crystallites. Top-view SEM images grown, with L = BDC **(a)**, Me-BDC **(b)**, MeO-BDC **(c)**. To show the coverage of the films, these micrographs display a larger area of the respective films shown in Figure 3, a-d, of the main manuscript. **(d)** The Zn_2L_2DABCO crystallites are characterized laterally by the length (L) and width (W), as schematically depicted, and their size was evaluated using ImageJ®. Notably, the length equals the height of the crystallites which is attributed to their crystal structure growing isostructural to Zn_2BDC_2DABCO, which itself has a tetragonal unit cell P4/mmm (see main manuscript).[1,2] Depending on the crystallite orientation, the long axis (crystal lattice b-direction) is oriented perpendicular to the substrate (face-up, (100) orientation), or parallel (face-down, (001) orientation).[3] **(e)** Platelet-like structure is grown for L = $(NH_2)_2$-BDC, where the lower Cu_2BDC_2 structure is evidenced in **(f)**......................... 9

Figure S3 Representative FT-IR spectra of the MOF structures grown. FT-IR spectra of the Cu_2BDC_2-on-$Cu(OH)_2$ substructure (turquoise trace) showing the asymmetric (v_{as}) and symmetric (v_{sy}) carboxylate vibrations located at 1569 cm^{-1} and 1398 cm^{-1}, respectively.[4] Conversion to the $Zn_2(NH_2)_2$-BDC_2DABCO system showed two vibrations named $v_{NH2\text{-}BDC}$ located at 1440 cm^{-1} and 1359 cm^{-1} attributed to the C-N vibrations of the amino-functionality in the BDC linker (orange trace). The weak mode at 1690 cm^{-1} indicates that the -COOH group is converted to $-COO^-$ for the functionalized BDC ligand,[5] thus its coordination to the zinc-metal nodes was successful. This is also found in the Zn_2BDC_2DABCO system (red trace). For the latter, strong vibrational bands ($v_{N\text{-}C\text{-}H}$) arising from the DABCO ligand are typically found around 750 cm^{-1} or 812 cm^{-1}.[2,6] However, these bands are missing for the $Zn_2(NH_2)_2$-BDC_2DABCO structure, which would indicate that the system is not grown following the pillared-layered motive yielding a 3D MOF system,[7] but rather as a two dimensional layer of $Zn_2(NH_2)_2$-BDC_2. Source data are provided as a Source Data file. 11

Figure S4 GIWAXS data and analysis. (a) Radial integration of GIWAXS pattern for the $Zn_2(NH_2)_2$-BDC_2DABCO structure grown for 90 min (purple trace) and 180 min (green trace) on oriented Cu_2BDC_2-on-$Cu(OH)_2$ films (red trace). **(b)** Out-of-plane and in-plane orientation of the films confirmed only the preferential alignment of the $(100)_{Cu2BDC2}$ reflection.[8,9] Closer inspection reveals an overlap of diffraction peaks that is attributed to the (100) reflection according to the crystal structures reported in ref. 6 (see red pattern, radial integration). The (200) reflection however shows no preferential alignment between the in-plane and out-of-plane direction. **(c)** Azimuthal angle scan (phi-scan) was conducted considering the in-plane direction to evaluate the preferential alignment of the reflections **(d)** $(100)_{Cu2BDC2}$ and (200) for the 180 min $Zn_2(NH_2)_2$-BDC_2DABCO film structure. The scan was performed in an angular range between 0 – 180°. **(e)** Polar plot of the $(100)_{Cu2BDC2}$ and the (200) reflections. **(f)** Results of the azimuthal angle scans of intensity profiles for the $(100)_{Cu2BDC2}$ and the (200) reflections of the film structure showing weak features of oriented alignment.[2] The highest intensity coincides close to 90 and 270°, indicating that the (200) reflection is oriented in-plane and parallel to the (100) reflection of the Cu_2BDC_2-on-$Cu(OH)_2$. It must be noted that the grown structure lacks the DABCO moiety as confirmed by infrared spectroscopic measurements resembling rather the $Zn_2(NH_2)_2$-BDC_2 film system (see Figure S3 and main manuscript text). Source data are provided as a Source Data file...12

Figure S5 Azimuthal GIWAXS scan analysis. Out-of-plane, in-plane and radial integration of the GIWAXS pattern for **(a)** Zn_2BDC_2DABCO **(b)** Zn_2MeO-BDC_2DABCO and **(c)** Zn_2Me-BDC_2DABCO. In-plane integrated GIWAXS pattern of the (100), (001), (110) or (101) reflections for Zn_2L_2DABCO and of the (001) reflection for the Cu_2BDC_2 substructure as a function of the rotation angle φ (azimuthal angle). The film rotation was conducted between 0

– 270°, for **(d)** Zn_2BDC_2DABCO, **(e)** Zn_2MeO-BDC_2DABCO, **(f)** Zn_2Me-BDC_2DABCO. Based on these GIWAXS data, the orientation of the films was deduced, and the preferential alignment of the upper Zn_2L_2DABCO structure with respect to the underlying Cu_2BDC_2-on-$Cu(OH)_2$ systems is displayed in the schematic in **(g).** Source data are provided as a Source Data file. 15

Figure S6 Evaluation of the degree of orientation using the GIXSGUI package.[14] (a) 2D GIWAXS pattern of the Zn_2MeO-BDC_2DABCO (incident angle 0.3°). The curved arrow is indicating the direction of azimuthal χ integration, where the wedge is located at χ = 0°. **(b)** The intensity distribution of the (100) reflection is shown where grey area indicates the contribution of the isotropic fraction of the crystallites. The 0° orientation corresponds to crystallites whose (100) planes are parallel to the substrate (FWHM = 22°; see main text, Figure 2b), whilst the crystallites itself grow perpendicular to the substrate. The degree of orientation (DO)[3] accounting for 80% was evaluated according to the details provided previously in ref. 2. Briefly, the DO (in %) is given by $Atotal - AisotropicAtotal$, where $Atotal$ denotes the area below the integrated intensity as a function of χ and $Aisotropic$ accounts for the isotropic fraction indicated by the grey area. The 95% confidence interval is provided as the shaded in the graph. **(c)** 2D GIWAXS pattern of the Zn_2Me-BDC_2DABCO film structure where the DO was estimated with 72% (FWHM = 34°) based on the intensity distribution of the (100) reflection shown in **(d)**. The 95% confidence interval is provided as the shaded in the graph. The structure grows isostructural to Zn_2MeO-BDC_2DABCO following its preferred growth of crystallites perpendicular to the substrate. Source data are provided as a Source Data file. 17

Figure S7 Uptake of CO_2 by the Zn-MOF film systems measured by QCM-D. All samples were purged prior the measurements with nitrogen for a smooth baseline. The CO_2 uptake/release was initiated at times indicated by the arrows. The mass was continuously monitored (Δf_3) and the mass change was calculated according to the Sauerbrey equation, with $\Delta m \sim C \cdot \Delta f$ (C = 17.7 ng cm^{-2} Hz^{-1}),[16] normalized to the mass of the MOF on the surface. **(a)** The bare QCM crystal (black trace) and upon grafting the Cu_2BDC_2 (blue trace) sub-structure show a very weak CO_2 adsorption. Growing the subsequent Zn-MOF layer show a significant increase in CO_2 uptake with **(b)** 0.14 ± 0.01 µg/cm² for Zn_2BDC_2DABCO, **(c)** 0.46 ± 0.03 µg/cm² for Zn_2Me-BDC_2DABCO and **(d)** 0.54 ± 0.03 µg/cm² for Zn_2MeO-BDC_2DABCO, evaluated after changes in the mass uptake reached saturation. The lightly shaded areas on the data points in **(a)** - **(d)** correspond to the 95% confidence interval based on 3 measurements. The transient response of the signal likely reflects pressure changes when switching between N_2 (CO_2 ON) and CO_2 (CO_2 OFF), affecting sensor oscillation. **(e)** Purging of Zn-MOF films and the sample compartment by N_2 was performed thoroughly to remove volatiles and establish a stable baseline for subsequent QCM-D measurements. **(f)** Sorption of CO_2 is fully reversible as evidenced by IR-spectromicroscopy measurements (different colours to highlight the five cycles of repeated CO_2 sorption). Source data are provided as a Source Data file. 20

Figure S8 Low-temperature IR spectroscopic study for Zn_2Me-BDC_2DABCO (left) and Zn_2MeO-BDC_2DABCO (right). Measurements were performed at 295 K (dashed spectra), 240 K (grey dotted spectra) and 200 K (black dash point spectra). Zoom-in of the spectra are shown for **(a)** the asymmetric (Δv_{as}) and **(b)** symmetric carboxylate mode (Δv_{sy}), **(c)** the deformation of the N-C-H moiety ($\Delta v_{N\text{-}C\text{-}H}$) and **(d)** mode related to adsorbed CO_2 ($\Delta v_{CO2\text{-}ad}$). The indicated shifts were determined considering the spectrum at 295 K and 200 K (ΔT = 95 K), where (+) denotes a blue-shift, and (-) a red-shift (visualized by arrows). Source data are provided as a Source Data file. 22

Figure S9 Low-temperature IR spectroscopic study for Zn_2BDC_2DABCO. Measurements were performed at 295 K (red dashed spectra), 240 K (grey dotted spectra) and 200 K (black

dash point spectra). Zoom-in of the spectra are shown for **(a)** the asymmetric (Δv_{as}) and **(b)** symmetric carboxylate mode (Δv_{sy}) and **(c)** the mode related to adsorbed CO_2 ($\Delta v_{CO2\text{-}ad}$). The indicated shifts were determined considering the spectrum at 295 K and 200 K (ΔT = 95 K), where (+) denotes a blue-shift, and (-) a red-shift (visualized by arrows). Source data are provided as a Source Data file..23

Figure S10 Zoom-in on the FT-IR spectra for Zn_2MeO-BDC_2DABCO related to the -OCH_3 functionality. (a) Spectrum denotes a blue-shift of $\Delta v_{\text{-}OCH3}$ = +1.2 cm^{-1}, whilst **(b)** no significant changes were found at $\Delta v_{\text{-}CH3}$ = 1400 cm^{-1}. Source data are provided as a Source Data file.24

Figure S11 Zoom-in on the FT-IR spectra for Zn_2Me-BDC_2DABCO related to the -CH_3 functionality. (a) denotes the -CH_3 vibration attributed to the Me-BDC linker showing a slight modulation upon CO_2 exposure whilst, **(b)** the -C-H stretching vibrations indicates no significant changes. **(c)** Similarly, the mode at 1398 cm^{-1} remained silent. Source data are provided as a Source Data file..24

Figure S12 Zoom-in on the GIWAXS pattern of the Zn_2L_2DABCO films. (a) Zn_2MeO-BDC_2DABCO **(b)** Zn_2Me-BDC_2DABCO and **(c)** Zn_2BDC_2DABCO, integrated along the in-plane direction for the (100) and the (001) reflection. Small changes are only observed for Zn_2Me-BDC_2DABCO (see main text). Source data are provided as a Source Data file. 25

Figure S 13 Zoom-in on the spectral region related to the azobenzene modes. IR spectra show the relaxed state at **(a)** 450 nm, and upon **(b)** excitation by 365 nm. **(a)** For Zn_2Me-BDC_2DABCO (dashed line spectra), the trans-azobenzene mode is located at $v_{t\text{-}AB}$ = 686 cm^{-1} and the cis-azobenzene mode at $v_{C\text{-}AB}$ = 698 cm^{-1}. Zn_2MeO-BDC_2DABCO (solid line spectra) shows mainly the trans-azobenzene mode located at $v_{t\text{-}AB}$ = 688 cm^{-1}. Thus, the trans-conformer between the two film structures experiences a shift of $\Delta v_{t\text{-}AB}$ = 1.9 cm^{-1}, attributable to the different chemical environment. **(b)** Upon excitation by 365 nm, both structures show signals at $v_{C\text{-}AB}$ = 698 cm^{-1} and $v_{t\text{-}AB}$ = 687 cm^{-1}. The broadening of the peaks in the case of Zn_2MeO-BDC_2DABCO is indicative for disorder in the MOF structure. **(c)** Zn_2Me-BDC_2DABCO shows 21% of azobenzene photo-switching after the first cycle and 19% after the second. **(d)** Zn_2MeO-BDC_2DABCO shows 22% of azobenzene photo-switching after the first cycle and 9% after the second. This decrease is attributed to the functional groups present in the Zn-MOF structure, as this behaviour is not found in the non-functionalized system.[2] Source data are provided as a Source Data file..26

Figure S14 Infiltration of azobenzene (AB) into the Zn-MOF film structures. (a) The Zn_2Me-BDC_2DABCO structure (dotted line pattern) shows a shift by $\Delta q_{(100)}$ = 0.04 nm^{-1} upon incorporation of AB molecules (solid line pattern). This corresponds to a slight contraction of the crystal lattice by Δd = 0.08 Å. **(b)** The Zn_2MeO-BDC_2DABCO structure (dotted line pattern) experiences a stronger change after AB infiltration (solid line pattern) with $\Delta q_{(100)}$ = 0.11 nm^{-1} that corresponds to a contraction by Δd = 0.22 Å. This result strongly supports the increased flexibility when introducing the MeO-BDC_2 linker into the Zn-MOF film structure. **(c)** The Zn_2BDC_2DABCO structure (dotted line pattern) shows a shift by $\Delta q_{(100)}$ = 0.07 nm^{-1} towards larger d-spacing upon incorporation of AB molecules (solid line pattern) indicating that the structure expands. Source data are provided as a Source Data file....................................27

Figure S15 Uptake of CO_2 by the azobenzene infiltrated Zn-MOF film systems measured by QCM-D. All samples were purged prior the measurements with nitrogen for a smooth baseline. The CO_2 uptake/release was initiated at times indicated by the arrows. Comparison with the non-infiltrated structures resulted in a decrease in CO_2 adsorption by **(a)** Δm_{CO2} = 0.42 µg/cm² for Zn_2Me-BDC_2DABCO, **(b)** Δm_{CO2} = 0.50 µg/cm² for Zn_2MeO-BDC_2DABCO and **(c)** Δm_{CO2} = 0.11 µg/cm² for Zn_2BDC_2DABCO. These differences in adsorbed CO_2 are attributed mainly to the different linker functionalization. The QCM-D experiments were repeated 4 times and a STD for the films was achieved of Zn_2MeO-BDC_2DABCO/AB 0.04 ± 0.01 µg/cm², Zn_2Me-

BDC_2DABCO/AB 0.04 ± 0.05 µg/cm², Zn_2BDC_2DABCO/AB 0.03 ± 0.01 µg/cm². Source data are provided as a Source Data file..28

Figure S16 IR spectra of azobenzene infiltrated Zn_2BDC_2DABCO/AB film prior and after low-pressure CO_2 load. (a) Spectra show no significant structural response of the azobenzene infiltrated Zn_2BDC_2DABCO/AB film prior (solid line spectra) and upon low-pressure CO_2 load (dotted line spectra). The asterisk denotes the mode at 720 cm^{-1} ascribed to the interaction between azobenzene and CO_2. **(b)** Photo-stimulation causes azobenzene to isomerize, with 46% of molecules converting to the cis-conformer. Source data are provided as a Source Data file. 29

1. Linker solutions pH determination

The pH for the methanolic $H_2(NH_2)_2$-BDC and $H_2(OH)_2$-BDC linker solutions were determined, where the amino-functionalized BDC linker showed a pH at about 6, whilst for $H_2(OH)_2$-BDC we determined with pH ~ 3 – 4. As a reference, the solvent MeOH was tested with pH ~ 6 as well as the non-substituted H_2BDC linker with pH ~ 5 – 6.

2. Integration of GIWAXS pattern

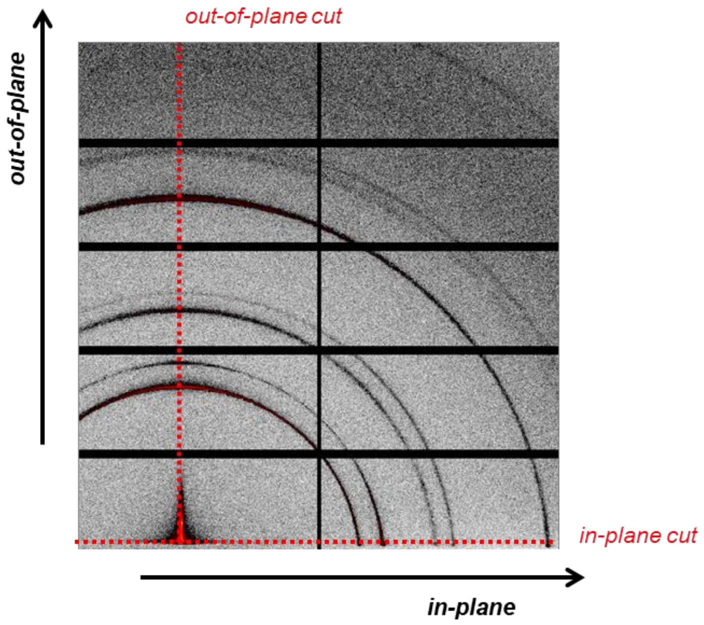

Figure S1 Sketch of GIWAXS data interpretation. Integration of 2D detector pattern was performed in the out-of-plane and the in-plane direction as denoted by the red dotted line, which corresponds to a cut margin of 20 pixels.

3. Photo-illumination protocol for monitoring the CO_2 uptake/release with QCM-D

The photo-illumination protocol was performed on the azobenzene infiltrated Zn_2L_2DABCO (L = Me-BDC, MeO-BDC) film systems. Initially, static mass values were obtained from QCM-D measurements (see Table S3, entries 1-2), along with in-situ CO_2 adsorption data for the Zn_2L_2DABCO/AB films (Figure S15). From these measurements, the CO_2 adsorption time was determined to be 8 minutes. This step was performed before photo-illumination, providing a baseline QCM-D measurement.

Subsequently, the films were illuminated first by 365 nm (*trans*-to-*cis* azobenzene isomerization) for 8 min under continuous CO_2 flow (0.1 L/min), controlled with calibrated flow controllers, followed by another static QCM-D measurement to assess the relative change in CO_2 adsorption. The process was then repeated with illumination at 451 nm (*cis*-to-*trans* azobenzene), completing one photo-illumination cycle. This protocol was repeated over four cycles, with the results discussed in the main manuscript. It is important to note that extended preconditioning of the Zn_2L_2DABCO films by purging with N_2 at 23°C is required. This step ensures a stable baseline, which is essential for accurate CO_2 uptake and release measurements.

4. SEM micrographs for Zn_2L_2DABCO film structures

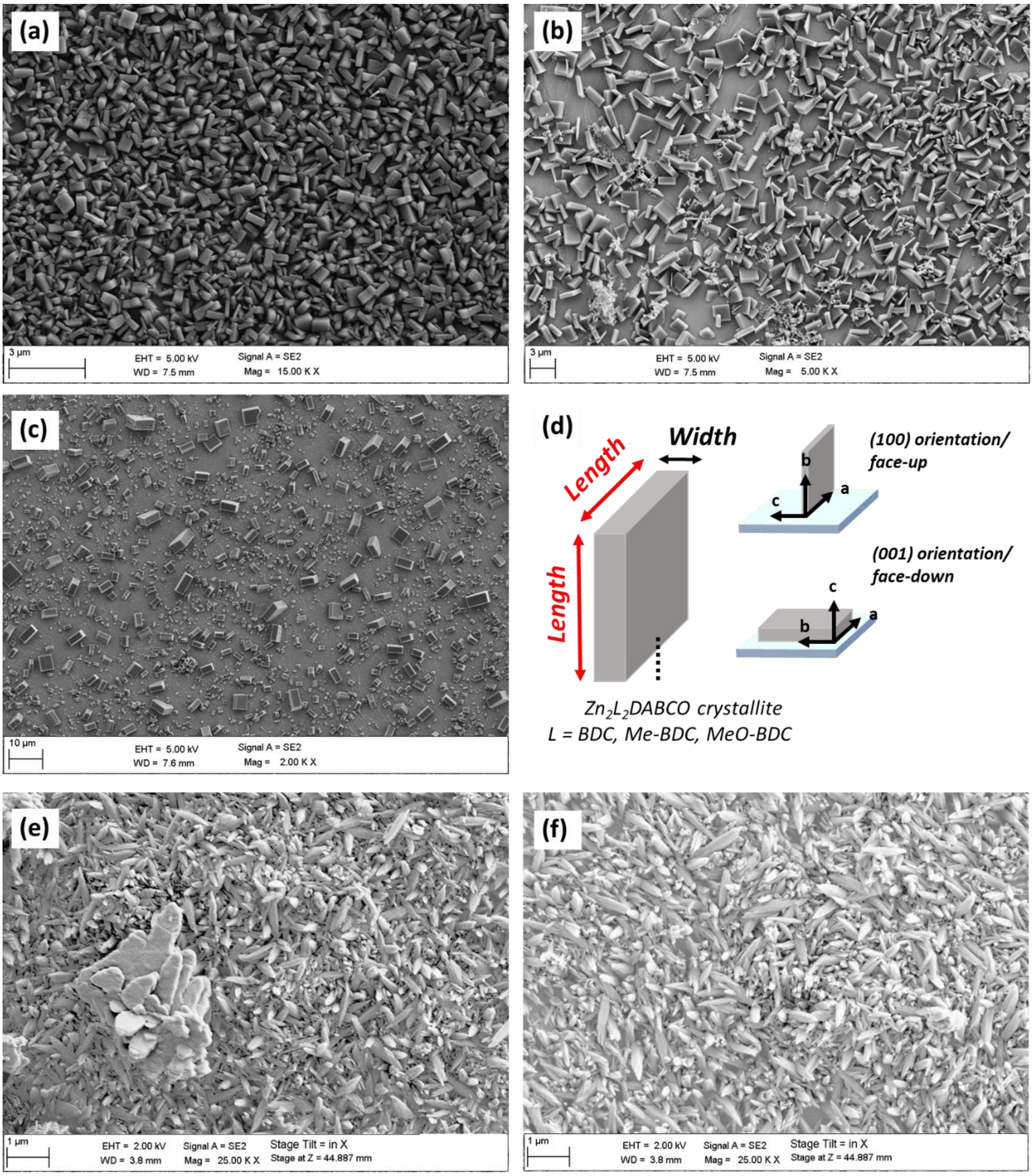

Figure S2 SEM images of the Zn_2L_2DABCO film structures and sketch of crystallites. Top-view SEM images grown, with L = BDC **(a)**, Me-BDC **(b)**, MeO-BDC **(c)**. To show the coverage of the films, these micrographs display a larger area of the respective films shown in Figure 3, a-d, of the main manuscript. **(d)** The Zn_2L_2DABCO crystallites are characterized laterally by the length (L) and width (W), as schematically depicted, and their size was evaluated using ImageJ®. Notably, the length equals the height of the crystallites which is attributed to their crystal structure growing isostructural to Zn_2BDC_2DABCO, which itself has a

tetragonal unit cell *P4/mmm* (see main manuscript).[1,2] Depending on the crystallite orientation, the long axis (crystal lattice *b*-direction) is oriented perpendicular to the substrate (face-up, (100) orientation), or parallel (face-down, (001) orientation).[3] **(e)** Platelet-like structure is grown for L = $(NH_2)_2$-BDC, where the lower Cu_2BDC_2 structure is evidenced in **(f)**.

5. IR and GIWAXS characterization of the $Zn_2(NH_2)_2$-BDC_2DABCO system

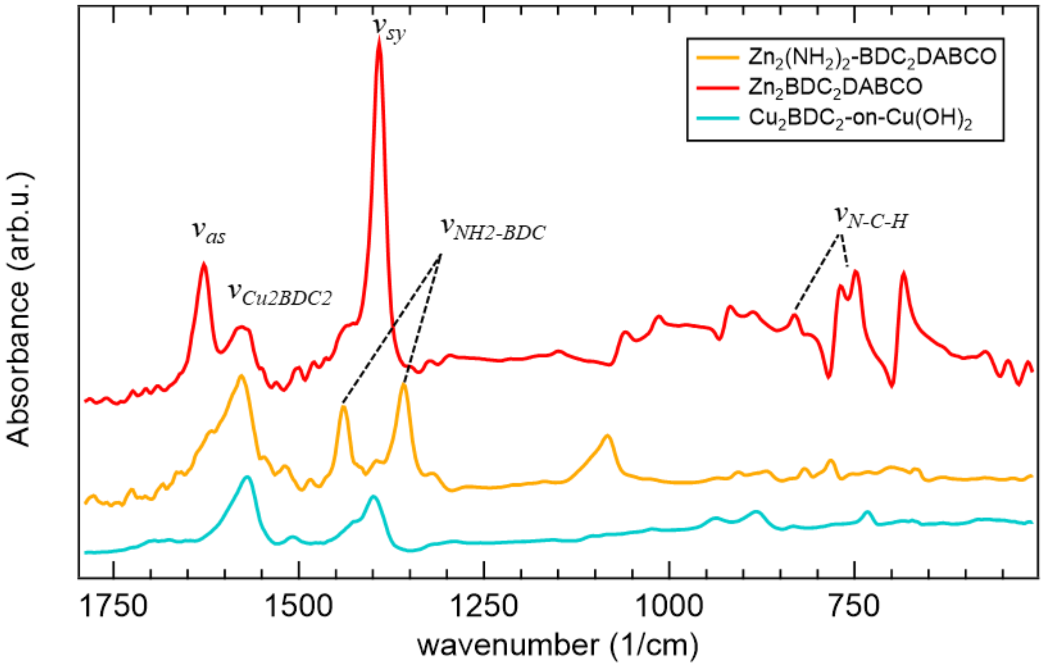

Figure S3 Representative FT-IR spectra of the MOF structures grown. FT-IR spectra of the Cu_2BDC_2-on-$Cu(OH)_2$ substructure (turquoise trace) showing the asymmetric (ν_{as}) and symmetric (ν_{sy}) carboxylate vibrations located at 1569 cm^{-1} and 1398 cm^{-1}, respectively.[4] Conversion to the $Zn_2(NH_2)_2$-BDC_2DABCO system showed two vibrations named $\nu_{NH2\text{-}BDC}$ located at 1440 cm^{-1} and 1359 cm^{-1} attributed to the C-N vibrations of the amino-functionality in the BDC linker (*orange trace*). The weak mode at 1690 cm^{-1} indicates that the -COOH group is converted to $-COO^-$ for the functionalized BDC ligand,[5] thus its coordination to the zinc-metal nodes was successful. This is also found in the Zn_2BDC_2DABCO system (red trace). For the latter, strong vibrational bands ($\nu_{N\text{-}C\text{-}H}$) arising from the DABCO ligand are typically found around 750 cm^{-1} or 812 cm^{-1}.[2,6] However, these bands are missing for the $Zn_2(NH_2)_2$-BDC_2DABCO structure, which would indicate that the system is not grown following the pillared-layered motive yielding a 3D MOF system,[7] but rather as a two dimensional layer of $Zn_2(NH_2)_2$-BDC_2. Source data are provided as a Source Data file.

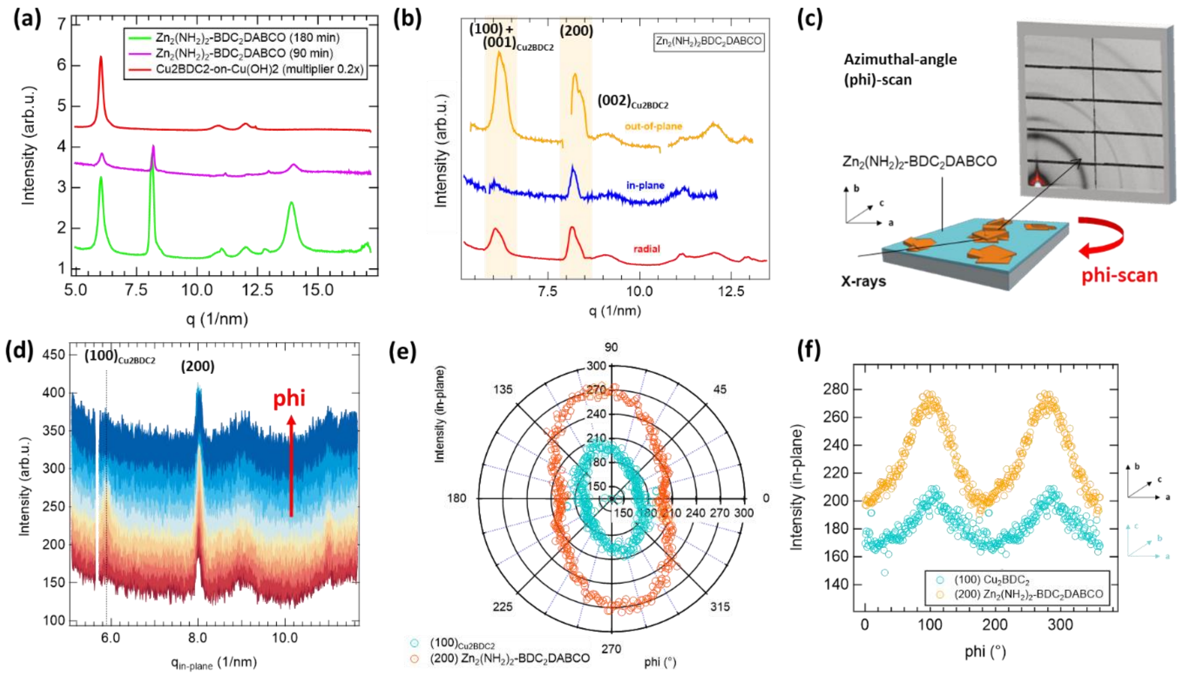

Figure S4 GIWAXS data and analysis. (a) Radial integration of GIWAXS pattern for the $Zn_2(NH_2)_2$-BDC_2DABCO structure grown for 90 min (purple trace) and 180 min (green trace) on oriented Cu_2BDC_2-on-$Cu(OH)_2$ films (red trace). **(b)** Out-of-plane and in-plane orientation of the films confirmed only the preferential alignment of the $(100)_{Cu2BDC2}$ reflection.[8,9] Closer inspection reveals an overlap of diffraction peaks that is attributed to the (100) reflection according to the crystal structures reported in ref. 6 (see red pattern, radial integration). The (200) reflection however shows no preferential alignment between the in-plane and out-of-plane direction. **(c)** Azimuthal angle scan (phi-scan) was conducted considering the in-plane direction to evaluate the preferential alignment of the reflections **(d)** $(100)_{Cu2BDC2}$ and (200) for the 180 min $Zn_2(NH_2)_2$-BDC_2DABCO film structure. The scan was performed in an angular range between 0 – 180°. **(e)** Polar plot of the $(100)_{Cu2BDC2}$ and the (200) reflections. **(f)** Results of the azimuthal angle scans of intensity profiles for the $(100)_{Cu2BDC2}$ and the (200) reflections of the film structure showing weak features of oriented alignment.[2] The highest intensity coincides close to 90 and 270°, indicating that the (200) reflection is oriented in-plane and parallel to the (100) reflection of the Cu_2BDC_2-on-$Cu(OH)_2$. It must be noted that the grown structure lacks the DABCO moiety as confirmed by infrared spectroscopic measurements resembling rather the $Zn_2(NH_2)_2$-BDC_2 film system (see Figure S3 and main manuscript text). Source data are provided as a Source Data file.

6. Isostructural growth of heteroepitaxial Zn_2L_2DABCO films (L = BDC, Me-BDC, MeO-BDC)

To evaluate the orientation of the Zn_2L_2DABCO structure with respect to the heteroepitaxial sublayer consisting of Cu_2BDC_2 grown on $Cu(OH)_2$, GIWAXS measurements were performed to assess the in-plane and out-of-plane alignment of the crystalline lattices along with their azimuthal angle dependence over the entire film structure with the results provided in Figure S5. To this aim, it is also important to summarize earlier findings on the epitaxial alignment of the Cu_2BDC_2 crystalline system with respect to the $Cu(OH)_2$ nanobelts, which have shown that the *a*-axis of Cu_2BDC_2 matches the *c*-axis of the sacrificial $Cu(OH)_2$ layer, and orthogonally to that the *b*-axis coincides with the *a*-axis, respectively.[8,9] Because of this epitaxial lattice match, the (100) reflection of Cu_2BDC_2 shows a strong azimuthal angle dependence that is in good agreement with the results obtained herein,[2,9] which further confirms that the epitaxial alignment of the sublayer persists even after the growth of the respective Zn_2L_2DABCO structures (Figure S5, a-c). The lattice match of the sublayers results in the *c*-axis of Cu_2BDC_2 being perpendicular to the substrate. Macroscopically, the Cu_2BDC_2 crystallites comprise a longer and shorter side (see schematics in Figure S5 e, blue triangular crystallites), which considering the lattice orientation results in their long side being parallel to the short-axis direction of $Cu(OH)_2$, and *vice versa*.[8,10]

The growth of the Zn_2L_2DABCO structures onto the Cu_2BDC_2-on-$Cu(OH)_2$ sublayer is directed by the lattice mismatch between these two lattice systems.[11] Considering those, the lowest lattice mismatch of about 1 – 3% is given when both *a*-axes are coinciding (**Table S 1** and **Table S 2**). Interestingly, this alignment is less pronounced for the Zn_2BDC_2DABCO structure, which shows a more preferential orientation in which the *a*-axis is aligned parallel to the *b*-axis of the Cu_2BDC_2 structure (and *c*-axis parallel to the *a*-axis, see Figure S5, d). As both the methyl and methoxy-functionalized Zn-MOF structure lack this flip in alignment, the absence of this orientation is attributed to the presence of bulkier functional groups, which could be directing the alignment of the Zn_2L_2DABCO structure. Hence, the functionalized structures remain also comparably more flexible because of their *c*-axis aligning along the non-

covalently interlinked *b*-axis of the Cu_2BDC_2 sublayer, whilst the Zn_2BDC_2DABCO structure remains more rigid because of its in-plane alignment flipped by 90°. It must be noted that the Zn_2L_2DABCO structures comprises also crystallites lying face down as evidenced by SEM micrographs (see Figure S2). This portion of crystallites cannot be unambiguously quantified by GIWAXS measurements, since the (0*k*0) plane is equivalent to the (*h*00) plane.[1]

The full width at half-maximum (fwhm) of the azimuthal angle dependence equals to the distribution of twisting angles of the Zn_2L_2DABCO crystallites along the in-plane direction, which eventually is influenced by the lattice mismatch ratio.[11] For the Zn_2L_2DABCO structures, the fwhm was found to increase by 6 – 14% with respect to the Cu_2BDC_2 sublayer, indicating a degradation of the in-plane orientation of the upper Zn_2L_2DABCO structures, which is related to the high lattice mismatch ratios.[11] Nevertheless, all three Zn_2L_2DABCO structures show epitaxial growth with respect to the Cu_2BDC_2-on-$Cu(OH)_2$ substructure along the entire substrate.

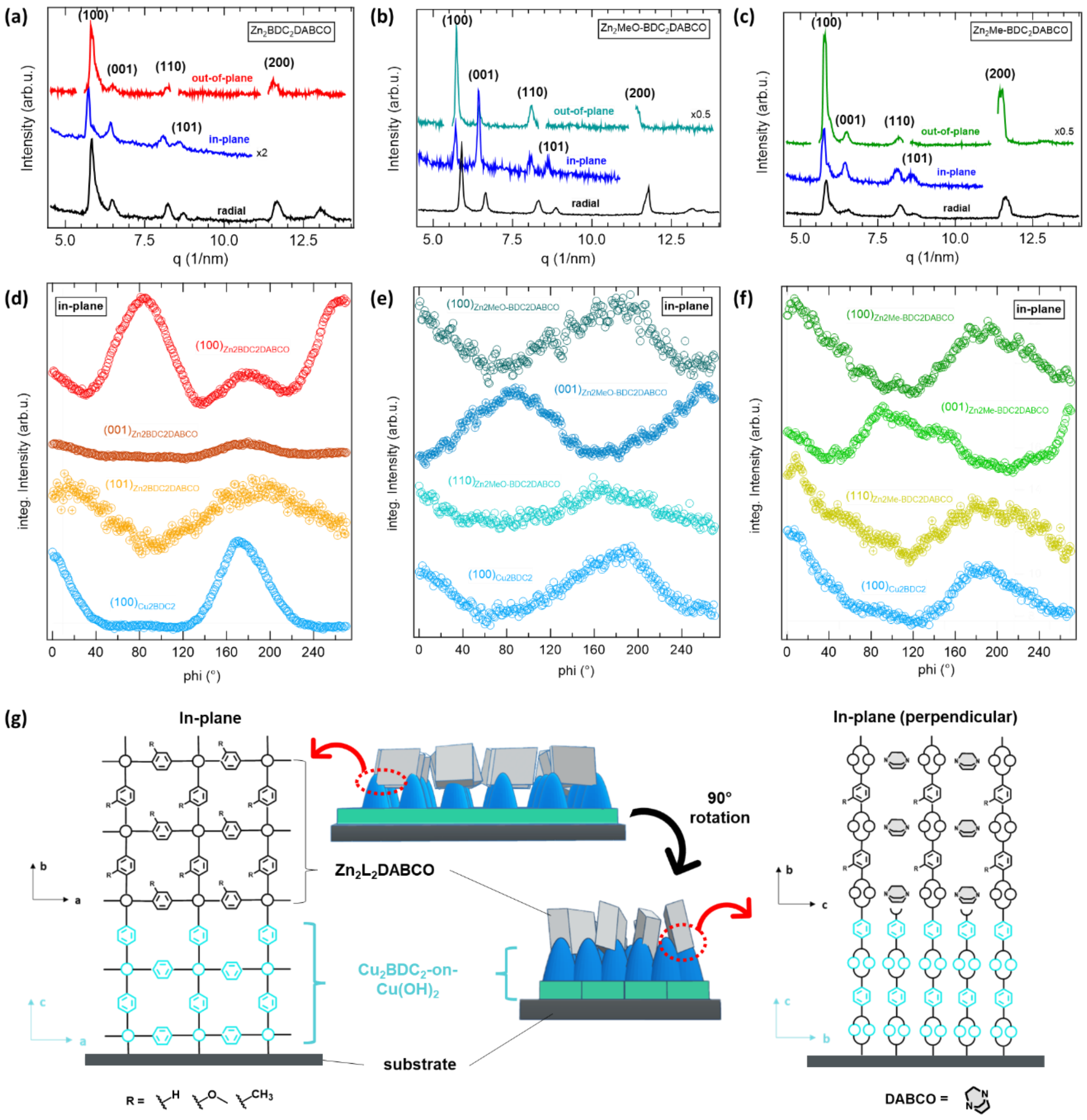

Figure S5 Azimuthal GIWAXS scan analysis. Out-of-plane, in-plane and radial integration of the GIWAXS pattern for **(a)** Zn_2BDC_2DABCO **(b)** Zn_2MeO-BDC_2DABCO and **(c)** Zn_2Me-BDC_2DABCO. In-plane integrated GIWAXS pattern of the (100), (001), (110) or (101) reflections for Zn_2L_2DABCO and of the (001) reflection for the Cu_2BDC_2 substructure as a function of the rotation angle φ (azimuthal angle). The film rotation was conducted between 0 – 270°, for **(d)** Zn_2BDC_2DABCO, **(e)** Zn_2MeO-BDC_2DABCO, **(f)** Zn_2Me-BDC_2DABCO. Based on these GIWAXS data, the orientation of the films was deduced, and the preferential alignment of the upper Zn_2L_2DABCO structure with respect to the underlying Cu_2BDC_2-on-$Cu(OH)_2$ systems is displayed in the schematic in **(g).** Source data are provided as a Source Data file.

Table S 1 Crystal lattice parameters for the investigated structures. The experimental lattice parameters of the epitaxial Zn-MOF films were evaluated from out-of-plane GIWAXS pattern.

(in Å)	Cu_2BDC_2[a]	Zn_2BDC_2DABCO (bulk)[b]	Zn_2BDC_2DABCO	Zn_2Me-BDC_2DABCO	Zn_2MeO-BDC_2DABCO
a	10.61	10.93	10.75	10.85	10.96
b	5.8	10.93	10.75	10.85	10.96
c	10.61	9.61	9.71	9.69	9.74

[a] Lattice parameter taken from ref. 8

[b] Lattice parameter taken from ref. 1

Table S 2 Calculated lattice mismatch ratio between the Zn-MOF crystalline lattice and the Cu_2BDC_2 substructure. The preferential alignment of the structure is anchored *via* the $a_{Zn\text{-}MOF}$-on-$a_{Cu2BDC2}$ and $c_{Zn\text{-}MOF}$-on-$b_{Cu2BDC2}$ directions (highlighted in grey).

	Calculated lattice mismatch (in %)			
	Zn_2BDC_2DABCO (bulk)	Zn_2BDC_2DABCO	Zn_2Me-BDC_2DABCO	Zn_2MeO-BDC_2DABCO
$a_{Zn\text{-}MOF}$ on $a_{Cu2BDC2}$	3.02	1.32	2.26	3.29
$c_{Zn\text{-}MOF}$ on $b_{Cu2BDC2}$	20.7	19.4	19.7	19.1
$c_{Zn\text{-}MOF}$ on $a_{Cu2BDC2}$	10.4	9.27	9.49	8.19
$a_{Zn\text{-}MOF}$ on $b_{Cu2BDC2}$	6.12	7.91	6.91	5.84

7. Orientation analysis of heteroepitaxial Zn-MOF films

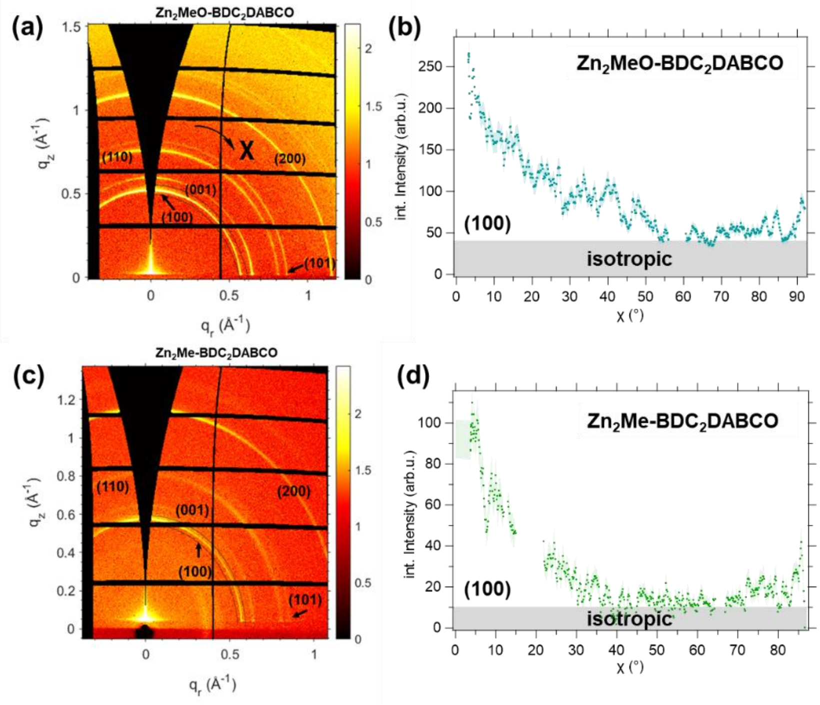

Figure S6 Evaluation of the degree of orientation using the GIXSGUI package.[14] (a) 2D GIWAXS pattern of the $Zn_2MeO-BDC_2DABCO$ (incident angle 0.3°). The curved arrow is indicating the direction of azimuthal χ integration, where the wedge is located at χ = 0°. **(b)** The intensity distribution of the (100) reflection is shown where grey area indicates the contribution of the isotropic fraction of the crystallites. The 0° orientation corresponds to crystallites whose (100) planes are parallel to the substrate (FWHM = 22°; see main text, Figure 2b), whilst the crystallites itself grow perpendicular to the substrate. The degree of orientation (*DO*)[3] accounting for 80% was evaluated according to the details provided previously in ref. 2. Briefly, the DO (in %) is given by $\frac{A_{total}-A_{isotropic}}{A_{total}}$, where A_{total} denotes the area below the integrated intensity as a function of χ and $A_{isotropic}$ accounts for the isotropic fraction indicated by the grey area. The 95% confidence interval is provided as the shaded in the graph. **(c)** 2D GIWAXS pattern of the $Zn_2Me-BDC_2DABCO$ film structure where the *DO* was estimated with 72% (FWHM = 34°) based on the intensity distribution of the (100) reflection shown in **(d)**. The 95% confidence interval is provided as the shaded in the graph. The structure grows isostructural to $Zn_2MeO-BDC_2DABCO$ following its preferred growth of crystallites perpendicular to the substrate. Source data are provided as a Source Data file.

8. CO_2 uptake by Zn_2L_2DABCO films (L = BDC, Me-BDC, MeO-BDC)

Static QCM-D measurements were performed for every layer of the heteroepitaxially grown film systems including $Cu(OH)_2$, Cu_2BDC_2 and the corresponding Zn_2L_2DABCO (L = BDC, Me-BDC, MeO-BDC). Prior to the measurement, the QCM-D cell was purged with N_2, and the mass of each layer was evaluated at room temperature (T = 23°C). These results are summarized in Table S3 (entries 1-2). Following this, the CO_2 uptake for every Zn-MOF film was assessed *via* QCM-D measurements during in-situ uptake studies, with the data provided in Figure S7. The relative mass change, corresponding to the adsorbed CO_2 within the MOF pores, was normalized to the mass of the empty Zn-MOF film before uptake (Table S3, entry 3).

To facilitate comparison with other MOF systems in literature, the adsorption capacity, typically expressed in mmol CO_2/g Zn-MOF, was calculated (Table S3, entries 4-5). As expected, with increasing polarity of the functional group, the adsorption capacity increased. In the case of the Zn-MOF films the type of response shown in Figure S7 indicates that only the MOF layer is highly sensitive towards CO_2. This is evidenced as a rapid increase in the response occurs due to the adsorption of the analyte caused by swelling of the Zn-MOF film and followed by a drop in response until the stabilization of the CO_2 adsorbed layer.[15] On a note, N_2 purging causes the desorption of residual solvents or other volatiles (i.e., MeOH, EtOH) from the films, which could be seen during the precondition of the samples represented by an increase in frequency shift (Figure S7, e). Because of this, the sample compartment was purged thoroughly to ensure a stable baseline. Also, N_2 itself is desorbed during CO_2 exposure, which explains the prolonged decrease of the signal in Figure 6e (main manuscript) and Figure S15. Upon N_2 exposure afterwards, CO_2 desorbs fully as evidenced by IR-spectromicroscopy measurements (see Figure S7, f), but the changed film structure does not allow N_2 or other volatiles to fill the same vacancies as before, causing a zero to negative net signal. From QCM-D measurements a CO_2 adsorption was achieved of Zn_2MeO-BDC_2DABCO/AB: 0.03 ± 0.01 µg/cm², Zn_2Me-BDC_2DABCO: 0.09 ± 0.05 µg/cm², Zn_2BDC_2DABCO: 0.04 ± 0.01 µg/cm².

Hence, Zn_2MeO-BDC_2DABCO adsorbs 6.7 times more CO_2 compared to the non-functionalized Zn_2BDC_2DABCO film system. After infiltrating the Zn-MOF films by azobenzene, a decrease in CO_2 adsorption capacity was observed, with the decreased quantity being influenced by the number of azobenzene molecules within the pores (see Table S4 for AB loading level).

Table S3 Results of gravimetric QCM-D measurements. Gravimetric QCM-D measurements of the respective Zn-MOF film layers grown heteroepitaxially on Cu_2BDC_2-on-$Cu(OH)_2$ films, and upon azobenzene (AB) infiltration demonstrating the successive increase in the total film mass. Evaluation of the CO_2 adsorption capacity of the Zn-MOF films.

	Entry 1	*Entry 2*	*Entry 3*	*Entry 4*	*Entry 5*
**System**	**Δf_3 (Hz)[a]**	**Mass (μg/cm^2)[a]**	**$\Delta Mass_{CO2}$ (μg/cm^2)[b]**	**$\Delta mmol_{CO2}$ /cm^2**	**Adsorption capacity (mmol CO_2/g Zn-MOF)**
$Cu(OH)_2$	606 ± 0.2	10.7	n.u.[c]	n.u.[c]	n.u.[c]
Cu_2BDC_2	929 ± 7	17.1	n.u.[c]	n.u.[c]	n.u.[c]
Zn_2BDC_2DABCO	2925 ± 16	51.4	0.14	3.18E-06	0.06
Zn_2BDC_2DABCO/AB	3137 ± 31	55.1	0.03	6.82E-07	0.01
difference			0.11	2.05E-06	0.05
Zn_2Me-BDC_2DABCO	3491 ± 37	61.2	0.46	1.05E-05	0.17
Zn_2Me-BDC_2DABCO/AB	3810 ± 4	67.4	0.04	9.09E-07	0.01
difference			0.42	9.54E-06	0.14
Zn_2MeO-BDC_2DABCO	1749 ± 5	30.9	0.54	1.23E-05	0.40
Zn_2MeO-BDC_2DABCO/AB	2077 ± 9	36.8	0.04	9.09E-07	0.02
difference			0.50	1.14E-05	0.31

[a] Mass uptake of CO_2 is evaluated from QCM-D results with the data provided in Figure S7.

[b] Static QCM-D measurements.

[c] No uptake

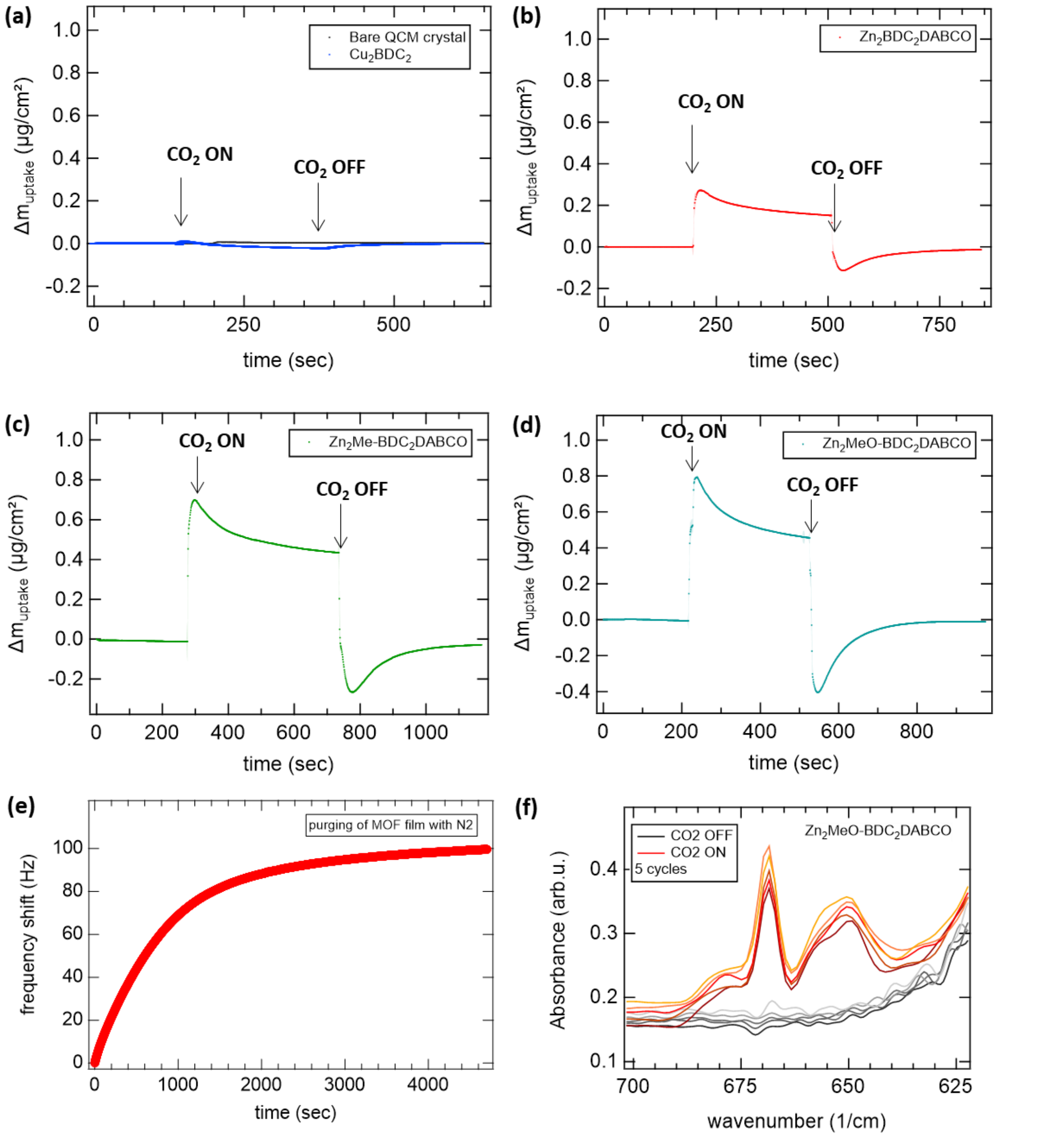

Figure S7 Uptake of CO_2 by the Zn-MOF film systems measured by QCM-D. All samples were purged prior the measurements with nitrogen for a smooth baseline. The CO_2 uptake/release was initiated at times indicated by the arrows. The mass was continuously monitored (Δf_3) and the mass change was calculated according to the Sauerbrey equation, with $\Delta m \sim C \cdot \Delta f$ ($C = 17.7$ ng cm^{-2} Hz^{-1}),[16] normalized to the mass of the MOF on the surface. **(a)** The bare QCM crystal (black trace) and upon grafting the Cu_2BDC_2 (blue trace) sub-structure show a very weak CO_2 adsorption. Growing the subsequent Zn-MOF layer show a significant increase in CO_2 uptake with **(b)** 0.14 ± 0.01 µg/cm² for Zn_2BDC_2DABCO, **(c)** 0.46 ± 0.03 µg/cm² for Zn_2Me-BDC_2DABCO and **(d)** 0.54 ± 0.03 µg/cm² for Zn_2MeO-BDC_2DABCO,

evaluated after changes in the mass uptake reached saturation. The lightly shaded areas on the data points in **(a) - (d)** correspond to the 95% confidence interval based on 3 measurements. The transient response of the signal likely reflects pressure changes when switching between N_2 (CO_2 ON) and CO_2 (CO_2 OFF), affecting sensor oscillation. **(e)** Purging of Zn-MOF films and the sample compartment by N_2 was performed thoroughly to remove volatiles and establish a stable baseline for subsequent QCM-D measurements. **(f)** Sorption of CO_2 is fully reversible as evidenced by IR-spectromicroscopy measurements (different colours to highlight the five cycles of repeated CO_2 sorption). Source data are provided as a Source Data file.

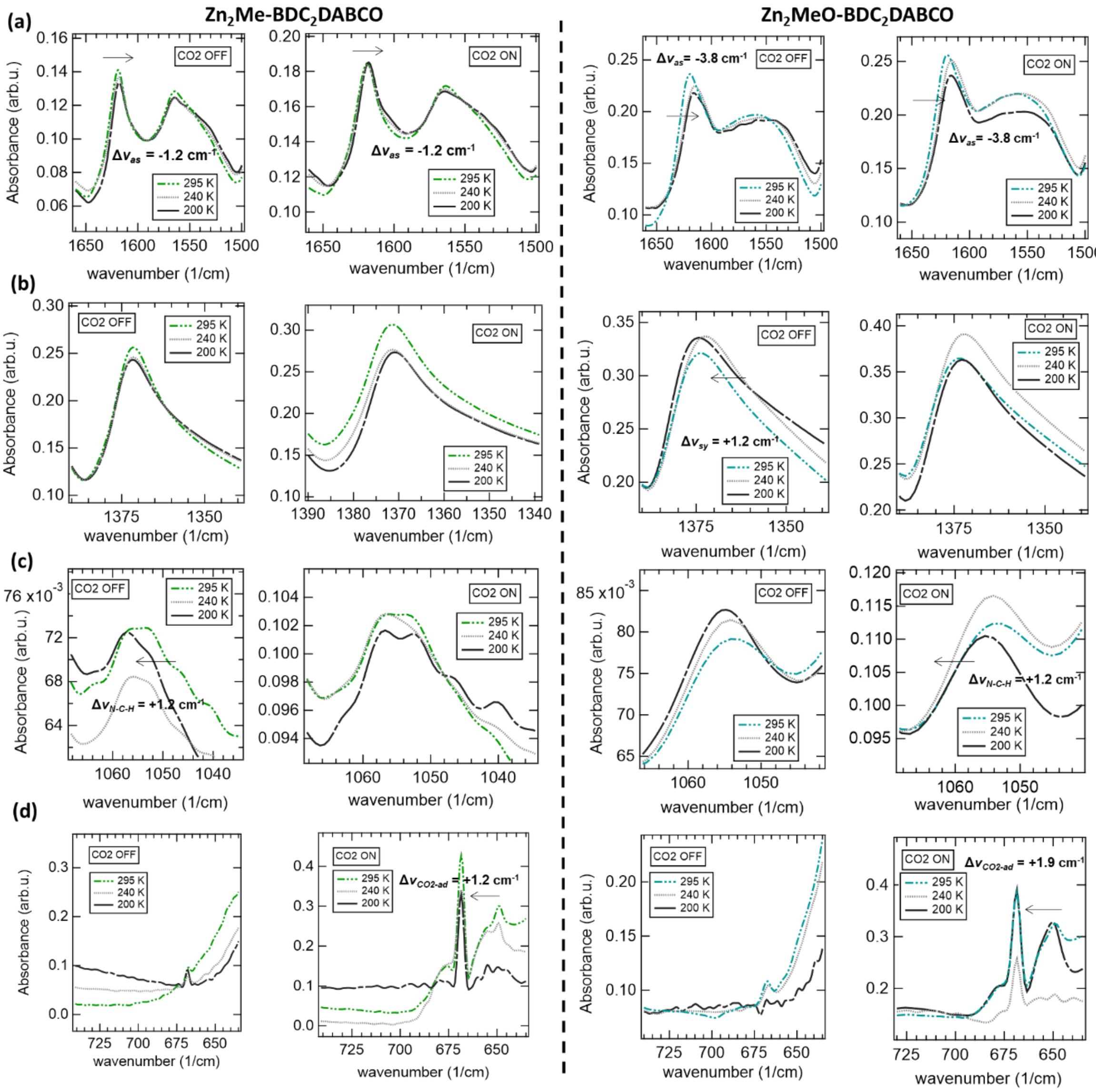

Figure S8 Low-temperature IR spectroscopic study for Zn_2Me-BDC_2DABCO (left) and Zn_2MeO-BDC_2DABCO (right). Measurements were performed at 295 K (dashed spectra), 240 K (grey dotted spectra) and 200 K (black dash point spectra). Zoom-in of the spectra are shown for **(a)** the asymmetric ($\Delta\nu_{as}$) and **(b)** symmetric carboxylate mode ($\Delta\nu_{sy}$), **(c)** the deformation of the *N-C-H* moiety ($\Delta\nu_{N\text{-}C\text{-}H}$) and **(d)** mode related to adsorbed CO_2 ($\Delta\nu_{CO2\text{-}ad}$). The indicated shifts were determined considering the spectrum at 295 K and 200 K (ΔT = 95 K), where (+) denotes a blue-shift, and (-) a red-shift (visualized by arrows). Source data are provided as a Source Data file.

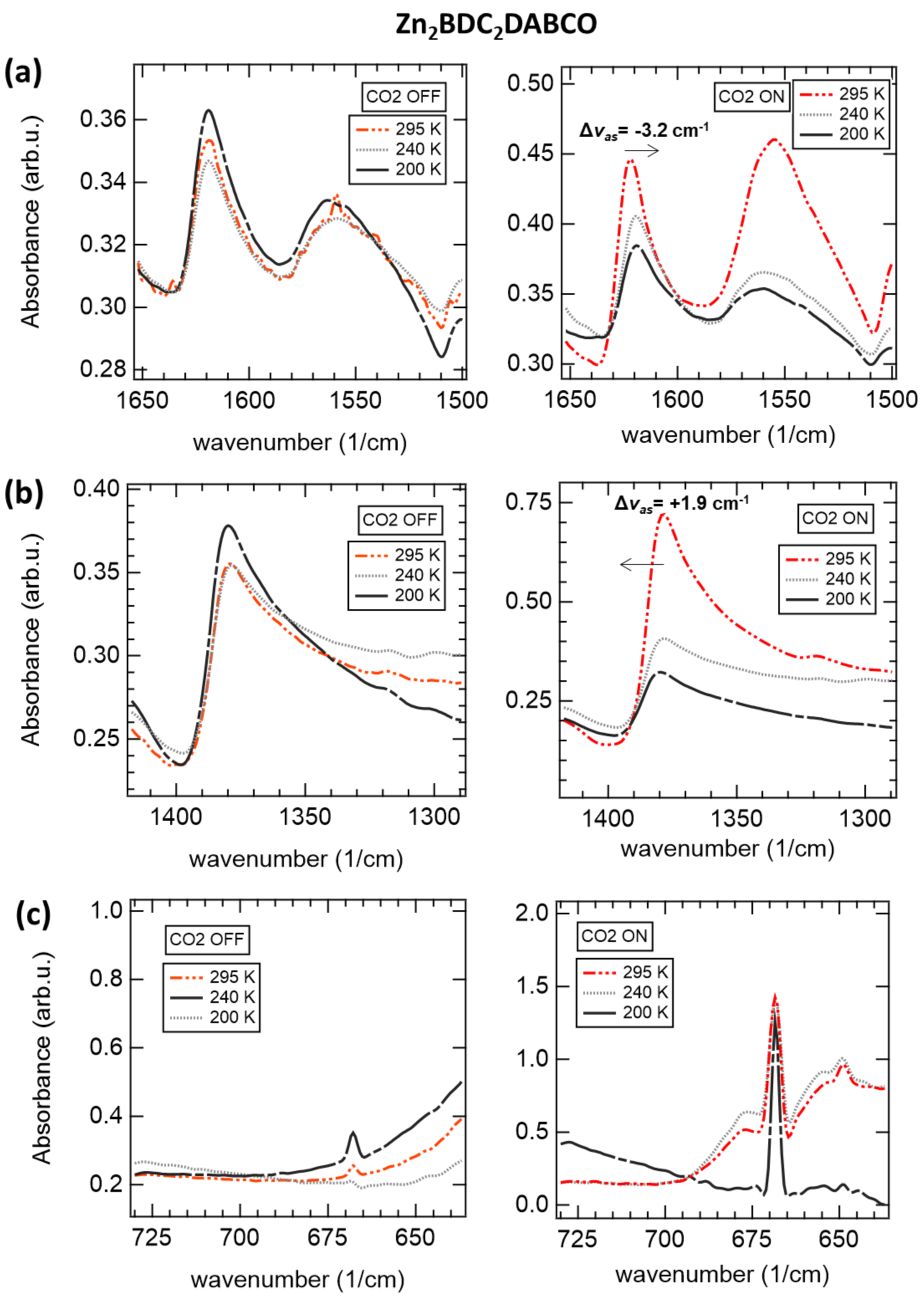

Figure S9 Low-temperature IR spectroscopic study for Zn_2BDC_2DABCO. Measurements were performed at 295 K (red dashed spectra), 240 K (grey dotted spectra) and 200 K (black dash point spectra). Zoom-in of the spectra are shown for **(a)** the asymmetric ($\Delta\nu_{as}$) and **(b)** symmetric carboxylate mode ($\Delta\nu_{sy}$) and **(c)** the mode related to adsorbed CO_2 ($\Delta\nu_{CO2\text{-}ad}$). The indicated shifts were determined considering the spectrum at 295 K and 200 K (ΔT = 95 K), where (+) denotes a blue-shift, and (-) a red-shift (visualized by arrows). Source data are provided as a Source Data file.

9. CO_2 uptake by Zn-MOF films (T = 295 K)

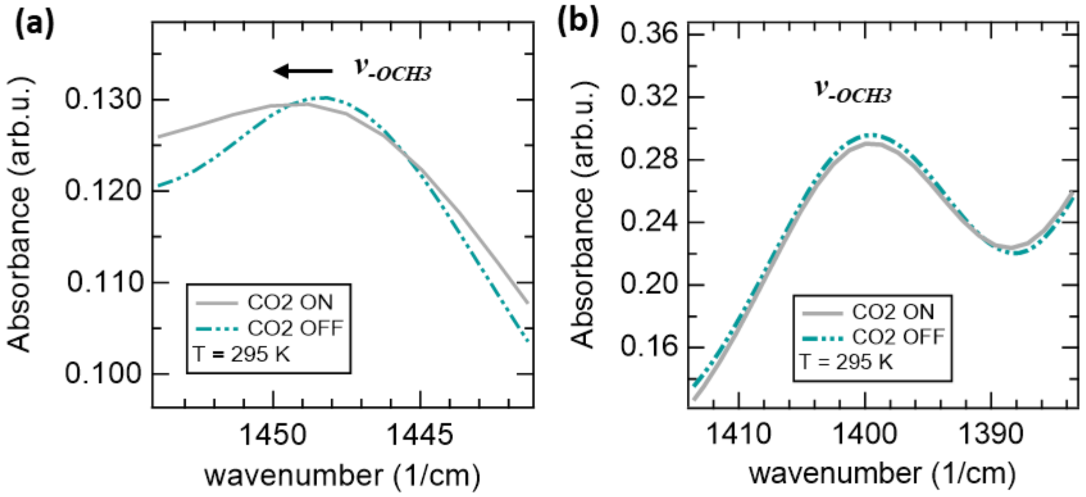

Figure S10 Zoom-in on the FT-IR spectra for Zn_2MeO-BDC_2DABCO related to the -OCH_3 functionality. (a) Spectrum denotes a blue-shift of $\Delta\nu_{\text{-OCH3}}$ = +1.2 cm^{-1}, whilst **(b)** no significant changes were found at $\Delta\nu_{\text{-CH3}}$ = 1400 cm^{-1}. Source data are provided as a Source Data file.

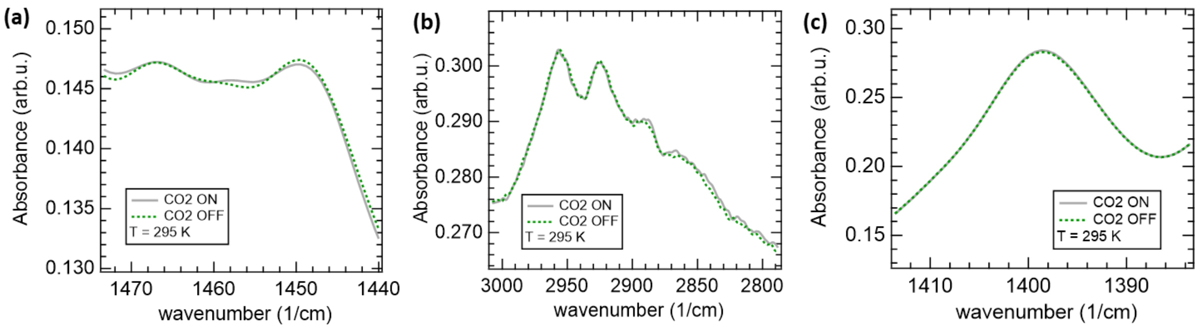

Figure S11 Zoom-in on the FT-IR spectra for Zn_2Me-BDC_2DABCO related to the -CH_3 functionality. (a) denotes the -CH_3 vibration attributed to the Me-BDC linker showing a slight modulation upon CO_2 exposure whilst, **(b)** the -C-H stretching vibrations indicates no significant changes. **(c)** Similarly, the mode at 1398 cm^{-1} remained silent. Source data are provided as a Source Data file.

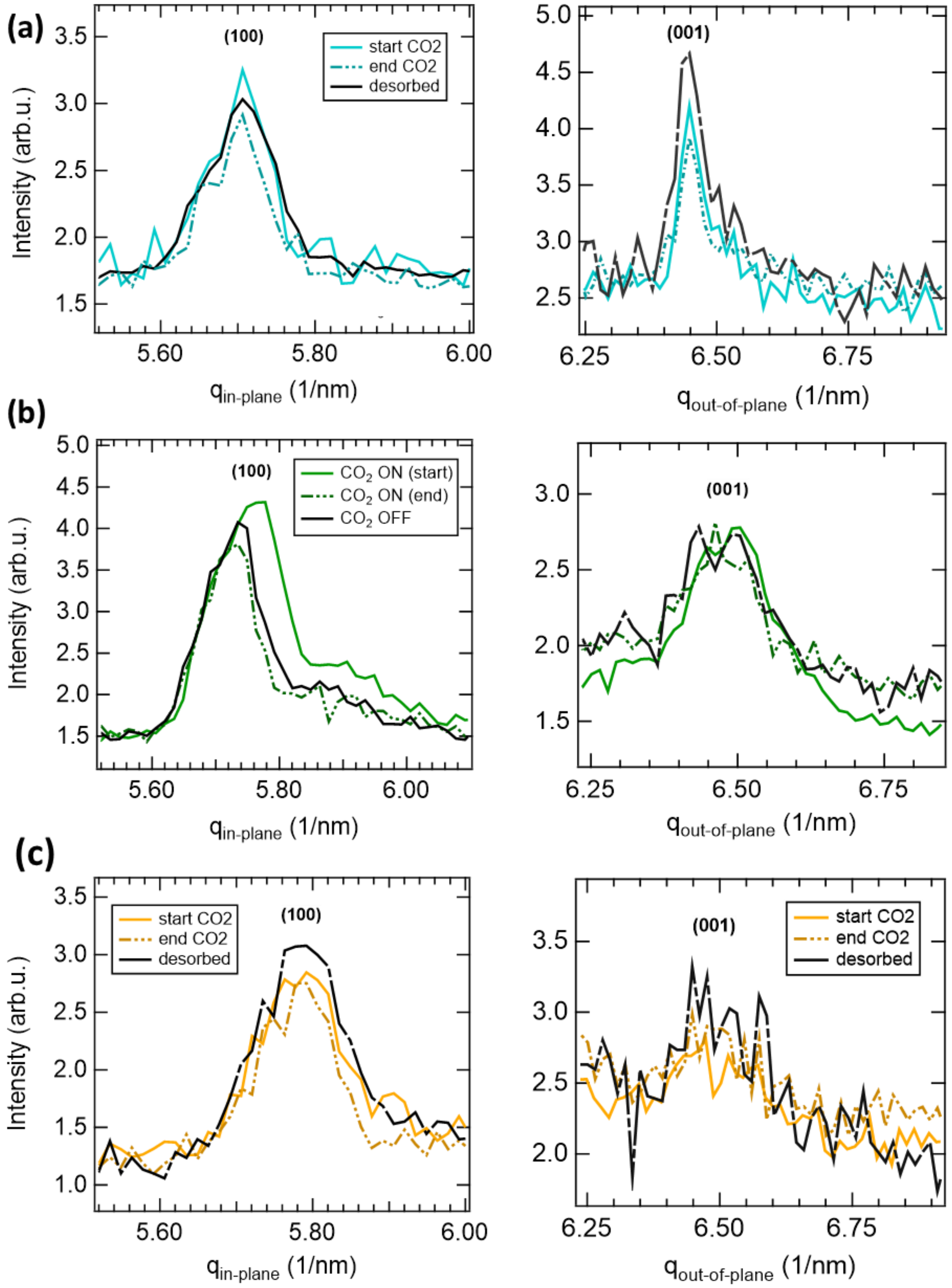

Figure S12 Zoom-in on the GIWAXS pattern of the Zn_2L_2DABCO films. (a) Zn_2MeO-BDC_2DABCO **(b)** Zn_2Me-BDC_2DABCO and **(c)** Zn_2BDC_2DABCO, integrated along the in-plane direction for the (100) and the (001) reflection. Small changes are only observed for Zn_2Me-BDC_2DABCO (see main text). Source data are provided as a Source Data file.

10. Azobenzene (AB) infiltration in Zn_2BDC_2DABCO, Zn_2MeO-BDC_2DABCO and Zn_2Me-BDC_2DABCO films

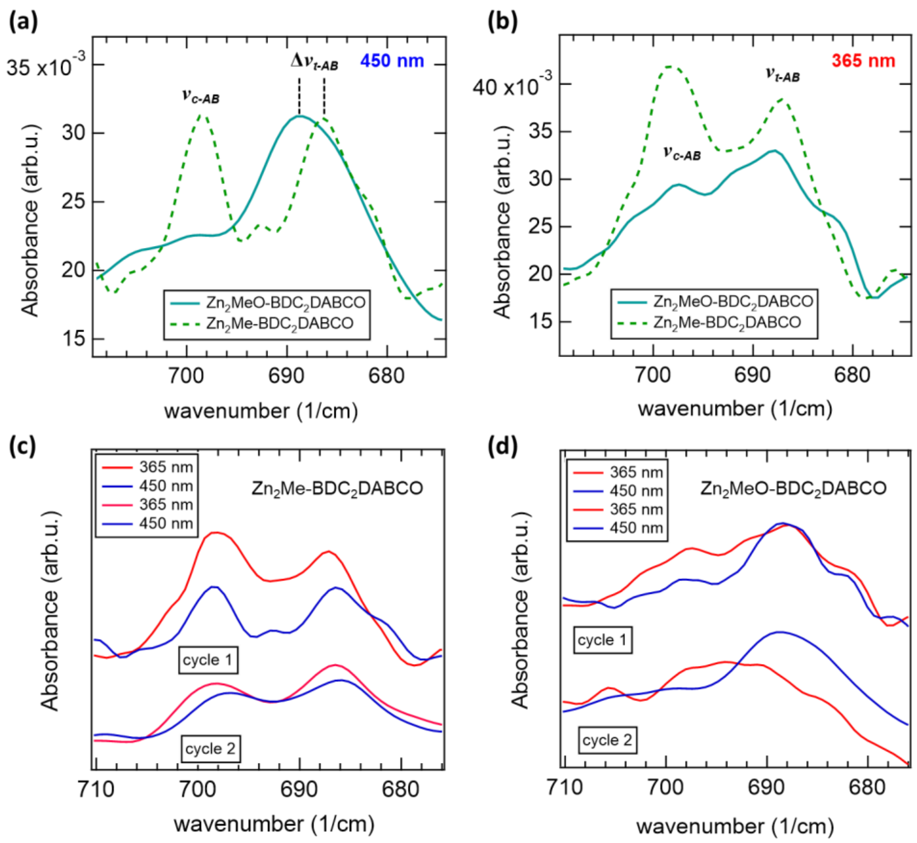

Figure S 13 Zoom-in on the spectral region related to the azobenzene modes. IR spectra show the relaxed state at **(a)** 450 nm, and upon **(b)** excitation by 365 nm. **(a)** For Zn_2Me-BDC_2DABCO (dashed line spectra), the *trans*-azobenzene mode is located at $\nu_{t\text{-}AB}$ = 686 cm^{-1} and the *cis*-azobenzene mode at $\nu_{C\text{-}AB}$ = 698 cm^{-1}. Zn_2MeO-BDC_2DABCO (solid line spectra) shows mainly the *trans*-azobenzene mode located at $\nu_{t\text{-}AB}$ = 688 cm^{-1}. Thus, the *trans*-conformer between the two film structures experiences a shift of $\Delta\nu_{t\text{-}AB}$ = 1.9 cm^{-1}, attributable to the different chemical environment. **(b)** Upon excitation by 365 nm, both structures show signals at $\nu_{C\text{-}AB}$ = 698 cm^{-1} and $\nu_{t\text{-}AB}$ = 687 cm^{-1}. The broadening of the peaks in the case of Zn_2MeO-BDC_2DABCO is indicative for disorder in the MOF structure. **(c)** Zn_2Me-BDC_2DABCO shows 21% of azobenzene photo-switching after the first cycle and 19% after the second. **(d)** Zn_2MeO-BDC_2DABCO shows 22% of azobenzene photo-switching after the first cycle and 9% after the second. This decrease is attributed to the functional groups present in the Zn-MOF structure, as this behaviour is not found in the non-functionalized system.[2] Source data are provided as a Source Data file.

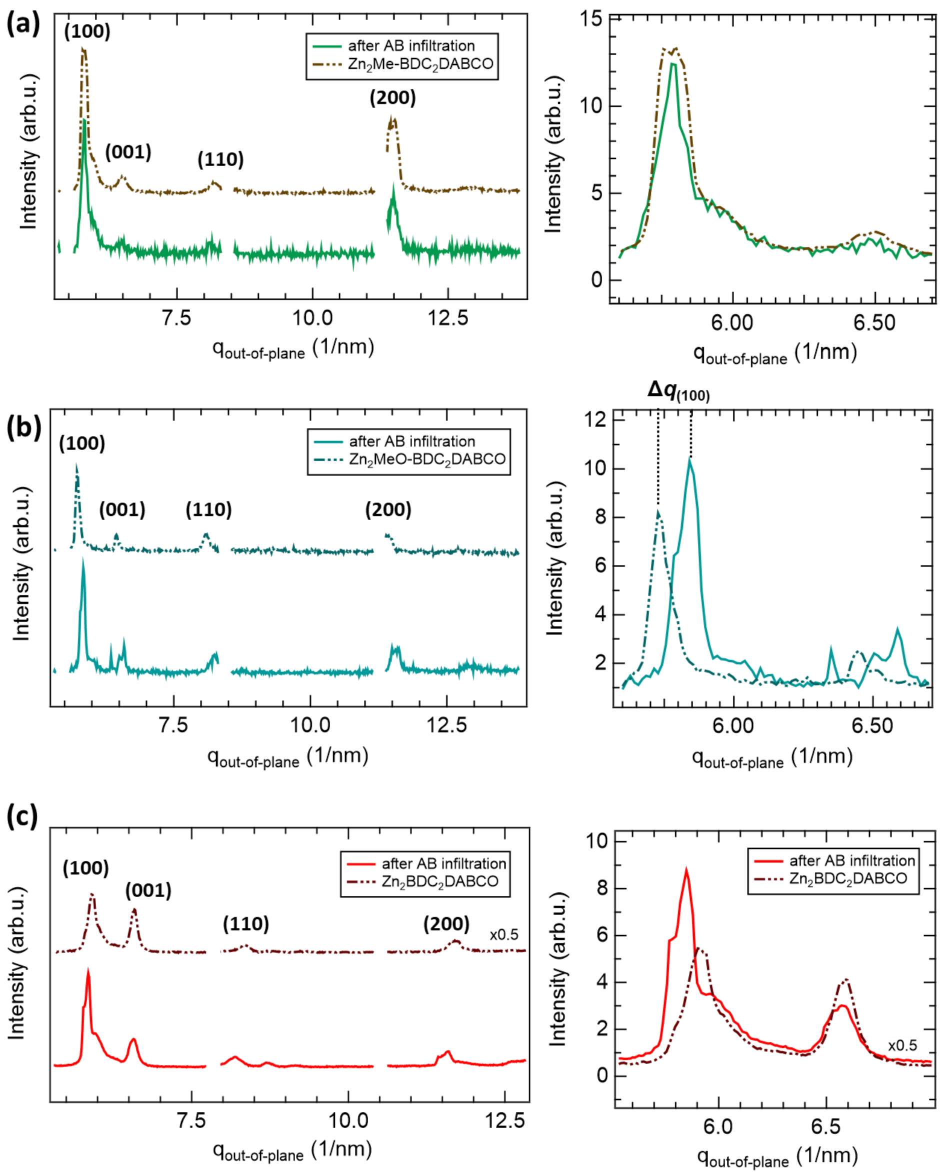

Figure S14 Infiltration of azobenzene (AB) into the Zn-MOF film structures. (a) The Zn_2Me-BDC_2DABCO structure (dotted line pattern) shows a shift by $\Delta q_{(100)}$ = 0.04 nm^{-1} upon incorporation of AB molecules (solid line pattern). This corresponds to a slight contraction of the crystal lattice by Δd = 0.08 Å. **(b)** The Zn_2MeO-BDC_2DABCO structure (dotted line pattern) experiences a stronger change after AB infiltration (solid line pattern) with $\Delta q_{(100)}$ = 0.11 nm^{-1} that corresponds to a contraction by Δd = 0.22 Å. This result strongly supports the increased flexibility when introducing the MeO-BDC_2 linker into the Zn-MOF film structure. **(c)** The Zn_2BDC_2DABCO structure (dotted line pattern) shows a shift by $\Delta q_{(100)}$ = 0.07 nm^{-1} towards larger *d*-spacing upon incorporation of AB molecules (solid line pattern) indicating that the structure expands. Source data are provided as a Source Data file.

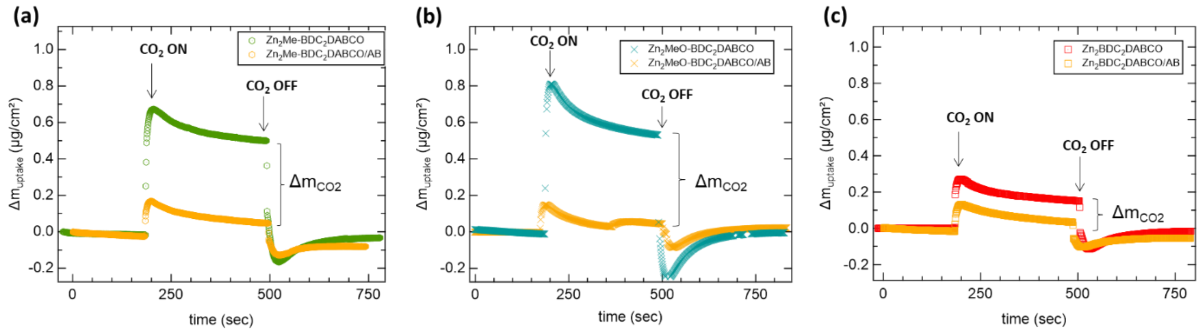

Figure S15 Uptake of CO_2 by the azobenzene infiltrated Zn-MOF film systems measured by QCM-D. All samples were purged prior the measurements with nitrogen for a smooth baseline. The CO_2 uptake/release was initiated at times indicated by the arrows. Comparison with the non-infiltrated structures resulted in a decrease in CO_2 adsorption by **(a)** Δm_{CO2} = 0.42 µg/cm² for $Zn_2Me\text{-}BDC_2DABCO$, **(b)** Δm_{CO2} = 0.50 µg/cm² for $Zn_2MeO\text{-}BDC_2DABCO$ and **(c)** Δm_{CO2} = 0.11 µg/cm² for Zn_2BDC_2DABCO. These differences in adsorbed CO_2 are attributed mainly to the different linker functionalization. The QCM-D experiments were repeated 4 times and a STD for the films was achieved of $Zn_2MeO\text{-}BDC_2DABCO$/AB 0.04 ± 0.01 µg/cm², $Zn_2Me\text{-}BDC_2DABCO$/AB 0.04 ± 0.05 µg/cm², Zn_2BDC_2DABCO/AB 0.03 ± 0.01 µg/cm². Source data are provided as a Source Data file.

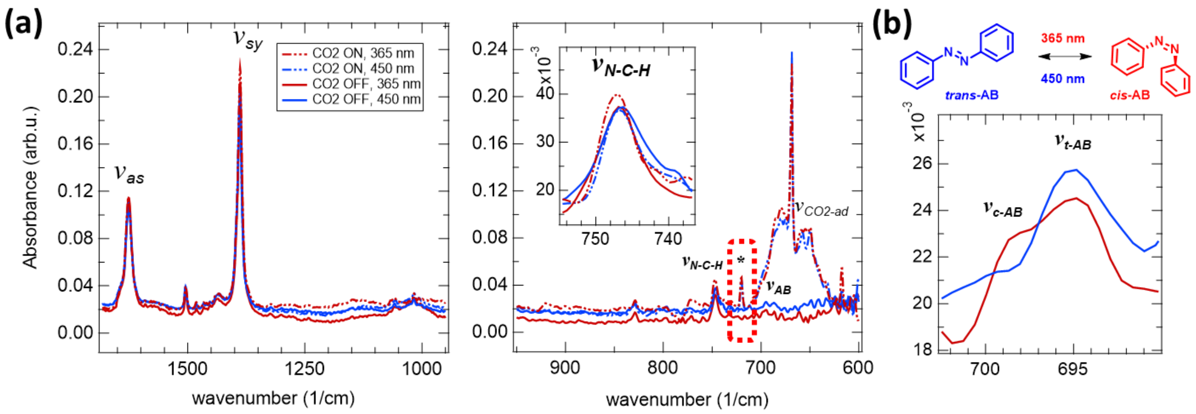

Figure S16 IR spectra of azobenzene infiltrated Zn_2BDC_2DABCO/AB film prior and after low-pressure CO_2 load. (a) Spectra show no significant structural response of the azobenzene infiltrated Zn_2BDC_2DABCO/AB film prior (solid line spectra) and upon low-pressure CO_2 load (dotted line spectra). The asterisk denotes the mode at 720 cm^{-1} ascribed to the interaction between azobenzene and CO_2. **(b)** Photo-stimulation causes azobenzene to isomerize, with 46% of molecules converting to the *cis*-conformer. Source data are provided as a Source Data file.

11. Calculation of AB loading level in Zn_2MeO-BDC_2DABCO and Zn_2OMe-BDC_2DABCO film structures

The loading level was calculated following the procedure reported in references 2 and 17, considering the number of AB molecules per Zn_2L_2DABCO pore (with L = Me- and MeO-BDC). The calculation was performed according to Eq. S1 and Eq. S2, with the relevant parameters summarized in Table S4. Owed to the broad size distribution of the Zn_2L_2DABCO crystallites, the AB loading level is given in a range, respectively.

$$Loading\left[\frac{molecules}{pore}\right] = c_{AB} * N_A * V_{\mathrm{Zn2L2DABCO}} \qquad \text{Eq. S1}$$

$$c_{AB}\left[\frac{mol}{L}\right] = \frac{\Delta A}{(\varepsilon_{cis-AB} - \varepsilon_{trans-AB}) * d_{\mathrm{Zn2L2DABCO}} * \Delta x_{AB}} \qquad \text{Eq. S2}$$

Table S4. Parameters for the calculation of the azobenzene loading in Zn_2L_2DABCO films (L = Me- and OMe-BDC). *ΔA* denotes the π-π* band, ε_{cis-AB} and $\varepsilon_{trans-AB}$ the extinction coefficients for the *cis*- and *trans*-isomers, respectively. Δx_{AB} refers to the change of the *cis*-content throughout the photo-switch determined from IR-spectroscopic measurements and $d_{Zn2L2DABCO}$ to the thickness of the Zn_2L_2DABCO crystallites oriented as the film system.

	ε_{cis-AB}	2 500 L/mol cm[8]
	$\varepsilon_{trans-AB}$	22 000 L/mol cm[8]
Zn_2Me-BDC_2DABCO	ΔA (λ = 330nm)	-0.0154
	Δx_{AB} [a]	0.13
	$d_{Zn2Me-BDC2DABCO}$ [b]	1.4 µm
	$V_{Zn2Me-BDC2DABCO}$ [c]	2281 Å³
	Loading level	~ 0.7 molecules/pore
Zn_2MeO-BDC_2DABCO	ΔA (λ = 330nm)	-0.009
	Δx_{AB} [a]	0.22
	$d_{Zn2MeO-BDC2DABCO}$ [b]	2 µm
	$V_{Zn2MeO-BDC2DABCO}$ [c]	2335 Å³
	Loading level	~ 0.2 molecules/pore

[a] Considering results from IR data

[b] The size of the crystallites was evaluated by ImageJ® displaying sizes in the denoted range. This parameter will influence the calculated loading level.

[c] Lattice parameters were deduced from the scattering pattern of *Zn_2L_2DABCO*

References

1. Dybtsev, D. N., Chun, H. & Kim, K. Rigid and flexible: a highly porous metal-organic framework with unusual guest-dependent dynamic behavior. *Angewandte Chemie (International ed. in English)* **43,** 5033–5036; 10.1002/anie.200460712 (2004).
2. Klokic, S. *et al.* Unraveling the timescale of the structural photo-response within oriented metal-organic framework films. *Chemical science* **13,** 11869–11877; 10.1039/d2sc02405e (2022).
3. Oesinghaus, L. *et al.* Toward Tailored Film Morphologies: The Origin of Crystal Orientation in Hybrid Perovskite Thin Films. *Adv Materials Inter* **3**; 10.1002/admi.201600403 (2016).
4. Stassin, T. *et al.* Vapour-phase deposition of oriented copper dicarboxylate metal-organic framework thin films. *Chemical communications (Cambridge, England)* **55,** 10056–10059; 10.1039/c9cc05161a (2019).
5. Tripathi, G. & Sheng, S. J. Solid-state vibrational spectra and structures of terephthalic acid and the terephthalate ion. *Journal of Molecular Structure* **57,** 21–34; 10.1016/0022-2860(79)80229-X (1979).
6. Xie, M., Prasetya, N. & Ladewig, B. P. Systematic screening of DMOF-1 with NH2, NO2, Br and azobenzene functionalities for elucidation of carbon dioxide and nitrogen separation properties. *Inorganic Chemistry Communications* **108,** 107512; 10.1016/j.inoche.2019.107512 (2019).
7. Senkovska, I. *et al.* Understanding MOF Flexibility: An Analysis Focused on Pillared Layer MOFs as a Model System. *Angewandte Chemie (International ed. in English)* **62,** e202218076; 10.1002/anie.202218076 (2023).
8. Falcaro, P. *et al.* Centimetre-scale micropore alignment in oriented polycrystalline metal-organic framework films via heteroepitaxial growth. *Nature materials* **16,** 342–348; 10.1038/nmat4815 (2017).
9. Linares-Moreau, M. *et al.* Semi-Automatic Deposition of Oriented Cu(OH) 2 Nanobelts for the Heteroepitaxial Growth of Metal–Organic Framework Films. *Adv Materials Inter* **8**; 10.1002/admi.202101039 (2021).
10. Klokic, S. *et al.* Orthogonal stimulation of structural transformations in photo-responsive MOF films through linker functionalization. *CrystEngComm* **26,** 2228–2232; 10.1039/D4CE00221K (2024).
11. Ikigaki, K., Okada, K. & Takahashi, M. Epitaxial Growth of Multilayered Metal–Organic Framework Thin Films for Electronic and Photonic Applications. *ACS Appl. Nano Mater.* **4,** 3467–3475; 10.1021/acsanm.0c03462 (2021).
12. Bristow, J. K., Butler, K. T., Svane, K. L., Gale, J. D. & Walsh, A. Chemical bonding at the metal–organic framework/metal oxide interface: simulated epitaxial growth of MOF-5 on rutile TiO 2. *J. Mater. Chem. A* **5,** 6226–6232; 10.1039/C7TA00356K (2017).
13. Pambudi, F. I., Anderson, M. W. & Attfield, M. P. Unveiling the mechanism of lattice-mismatched crystal growth of a core-shell metal-organic framework. *Chemical science* **10,** 9571–9575; 10.1039/c9sc03131f (2019).
14. Jiang, Z. GIXSGUI: a MATLAB toolbox for grazing-incidence X-ray scattering data visualization and reduction and indexing of buried three-dimensional periodic nanostructured films. *J Appl Crystallogr* **48,** 917–926; 10.1107/S1600576715004434 (2015).

15. Kruglenko, I. & Snopok, B. Analyte-Responsive Metal–Organic Frameworks of Polymer-Stabilized Silver Nanoparticles for Gas Sensors: A Comparative Study Using Surface Plasmon Resonance and Quartz Crystal Microbalance Techniques. In *ECSA-11* (MDPI, Basel Switzerland), p. 64.

16. Johannsmann, D. *The quartz crystal microbalance in soft matter research. An introduction to modeling and data analysis* (Springer, Berlin, 2014).

17. Koehler, T., Strauss, I., Mundstock, A., Caro, J. & Marlow, F. Reversible Photoalignment of Azobenzene in the SURMOF HKUST-1. *The journal of physical chemistry letters* **12,** 8903–8908; 10.1021/acs.jpclett.1c02489 (2021).
